# Correction: Ecological correlates of chimpanzee (*Pan troglodytes schweinfurthii*) density in Mahale Mountains National Park, Tanzania

**DOI:** 10.1371/journal.pone.0253673

**Published:** 2021-06-22

**Authors:** Adrienne B. Chitayat, Serge A. Wich, Matthew Lewis, Fiona A. Stewart, Alex K. Piel

There are errors in Table 3. Multiple values in Table 3 are incorrect. Please see the correct Table 3 here.

**Table 3:** Overview of important vegetation types found in MMNP and utilized by chimpanzees for nesting.

	Open-canopy forest	Closed-canopy forest	Miombo woodland	Bamboo woodland
***All species***				
No. of species	144	125	147	19
Diversity (Shannon Index)	4.2	3.9	3.8	2.4
Tree density/ha	97	358	190	5
Basal area/ha	5.3	23.8	9.0	0.1
***Feeding species***				
No. of species	56	47	59	10
Diversity (Shannon Index)	3.6	3.0	3.1	1.7
Tree density/ha	46	166	120	3
Basal area/ha	2.6	11.9	5.9	0.1
