# Supplementary material for: Ecological correlates of chimpanzee (Pan troglodytes schweinfurthii) density in Mahale Mountains National Park, Tanzania
Source: PLoS One. 2021 Feb 12;16(2):e0246628. doi: 10.1371/journal.pone.0246628 (PMC7880473; doi:10.1371/journal.pone.0246628)
Supplement: S1 Table — (DOCX) [file pone.0246628.s001.docx]

| **Family** | ***Genus species*** | **Site** | **Nesting** |
| --- | --- | --- | --- |
| Anacardiaceae | *Lannea schimperi* | M | ✓ |
|  | *Pseudospondias microcarpa* | M, G | ✓ |
| Anacardiaceae | *Rhus vulgaris* | G |  |
| Anisophylleaceae | *Anisophyllea boehmii* | G, I |  |
| Annonaceae | *Annona senegalensis* | M, G, I | ✓ |
|  | *Hexalobus monopetalus* | I |  |
|  | *Monanthotaxis poggei* | G, I |  |
|  | *Uvaria angolensis* | M, G, I |  |
|  | *Xylopia longipetala* | M | ✓ |
| Apocynaceae | *Tabernaemontana pachysiphon* | M |  |
| Boraginaceae | *Cordia africana* | M, G |  |
| Caesalpiniaceae | *Afzelia africana* | M |  |
|  | *Bauhinia petersiana* | M |  |
|  | *Piliostigma thonningii* | M, G, I |  |
| Chrysobalanaceae | *Parinari curatellifolia* | M, G, I | ✓ |
| Combretaceae | *Combretum molle* | M, G | ✓ |
|  | *Combretum* sp. | I |  |
| Ebenaceae | *Diospyros kirkii* | M |  |
| Euphorbiaceae | *Antidesma venosum* | G, I |  |
|  | *Croton sylvaticus* | M, G |  |
|  | *Margaritaria discoidea* | M |  |
|  | *Pseudolachnostylis maprouneifolia* | I |  |
|  | *Ricinodendron heudelotii* | M |  |
|  | *Uapaca kirkiana* | M, G, I |  |
|  | *Bridelia micrantha* | M, G | ✓ |
|  | *Hymenocardia acida* | G |  |
| Fabaceae | *Bauhinia thonningii* | I |  |
|  | *Brachystegia boehmii* | I | ✓ |
|  | *Brachystegia bussei* | M, G, I | ✓ |
|  | *Brachystegia longifolia* | G | ✓ |
|  | *Brachystegia spiciformis* | M, I | ✓ |
|  | *Julbernardia globiﬂora* | I | ✓ |
|  | *Julbernardia unijugata* | I | ✓ |
|  | *Pterocarpus angolensis* | M, G | ✓ |
|  | *Pterocarpus tinctorius* | M, G, I | ✓ |
| Flacourtiaceae | *Flacourtia indica* | M, I |  |
| Gramineae | *Elaeis guineensis* | G |  |
| Guttiferae | *Garcinia huillensis* | M, G, I | ✓ |
|  | *Harungana madagascariensis* | M, G |  |
| Loganiaceae | *Strychnos innocua* | M, G, I |  |
|  | *Strychnos cocculoides* | G, I |  |
| Malvaceae | *Thespesia garckeana* | I |  |
| Mimosaceae | *Acacia hockii* | M, G |  |
|  | *Albizia glaberrima* | M, G |  |
|  | *Entada abyssinica* | G |  |
|  | *Newtonia buchananii* | G |  |
| Moraceae | *Ficus congensis* | M, G |  |
|  | *Ficus cyathistipula* | M |  |
|  | *Ficus exasperata* | M, G, I |  |
|  | *Ficus* sp. | M, G, I |  |
|  | *Ficus thonningii* | M, G | ✓ |
|  | *Myrianthus holstii* | M |  |
|  | *Milicia excelsa* | M |  |
| Myristicaceae | *Pycnanthus angolensis* | M, G |  |
| Myrtaceae | *Syzygium guineense* | M, G, I | ✓ |
| OIacaceae | *Ximenia americana* | M |  |
|  | *Ximenia caffra* | I |  |
|  | *Schrebera alata* | G |  |
| Papilionaceae | *Neorautanenia mitis* | M, G |  |
|  | *Erythrina abyssinica* | M |  |
| Phyllanthaceae | *Uapaca nitida* | M, G, I |  |
| Protaceae | *Protea welwitschii* | G |  |
| Rhamnaceae | *Ziziphus abyssinica* | I |  |
| Rubiaceae | *Canthium rubrocostatum* | M |  |
|  | *Keetia gueinzii* | I |  |
|  | *Oxyanthus speciosus* | I |  |
|  | *Psychotria* sp. | I |  |
|  | *Rothmannia manganjae* | M |  |
|  | *Tricalysia coriacea* | I |  |
| Sapindaceae | *Allophylus congolanus* | M, I |  |
|  | *Allophylus* sp. | M, G |  |
|  | *Lecaniodiscus fraxinifolius* | M |  |
|  | *Zanha africana* | I |  |
| Sapotaceae | *Afrosersalisia cerasifera* | M, G |  |
| Sterculiaceae | *Dombeya rotundifolia* | M, G |  |
|  | *Sterculia quinqueloba* | M, G | ✓ |
|  | *Sterculia tragacantha* | M, G |  |
|  | *Sterculia* sp. | M, G |  |
| Tiliaceae | *Grewia mollis* | M, G |  |
| Ulmaceae | *Celtis africana* | M | ✓ |
|  | *Trema orientalis* | M |  |
| Umbelliferae | *Steganotaenia araliacea* | G |  |
| Verbenaceae | *Vitex doniana* | M, G, I |  |
